# Effect of Korean Herbal Medicine Combined with a Probiotic Mixture on Diarrhea-Dominant Irritable Bowel Syndrome: A Double-Blind, Randomized, Placebo-Controlled Trial

**DOI:** 10.1155/2013/824605

**Published:** 2013-12-05

**Authors:** Seok-Jae Ko, Gajin Han, Seul-Ki Kim, Jae-Gu Seo, Won-Seok Chung, Bongha Ryu, Jinsung Kim, Inkwon Yeo, Beom-Joon Lee, Jin-Moo Lee, Jae-Woo Park

**Affiliations:** ^1^College of Korean Medicine, Kyung Hee University, 26 Kyungheedae-ro, Dongdaemun-gu, Seoul 130-701, Republic of Korea; ^2^Cell Biotech, Co., Ltd., Gaegok-Ri, Wolgot-myeon, Gyeonggi-do, Gimpo-si 415-871, Republic of Korea; ^3^College of Sookmyung Women's University, Cheongpa-dong 2-ga, Yongsan-gu, Seoul 140-742, Republic of Korea; ^4^Kangnam Korean Hospital, Kyung Hee University, 26 Kyungheedae-ro, Dongdaemun-gu, Seoul 130-701, Republic of Korea

## Abstract

*Introduction*. Although combination therapy with herbal medicine and probiotics is gaining popularity for controlling diarrhea-dominant irritable bowel syndrome (D-IBS) symptoms, few studies have investigated its clinical effects. *Materials and Methods*. Fifty-three patients with D-IBS were randomly allocated into 1 of the following 4 groups: herbal medicine (*Gwakhyangjeonggisan*; GJS) plus probiotics (Duolac7S; DUO), GJS plus placebo DUO, placebo GJS plus DUO, and placebo GJS plus placebo DUO. The study period consisted of a 2-week run-in, 8 weeks of administration, and 2 weeks of follow-up. The primary outcomes were weekly adequate relief (AR) of overall IBS symptoms and the proportion of responders (PR) during the administration period. The secondary outcomes included individual IBS symptoms, stool assessment, and quality of life. Changes of intestinal microbiota and intestinal permeability were also analyzed. *Results and Discussion*. Weekly AR was not different among the 4 groups throughout the treatment period. However, the 3 treatment groups exhibited significant improvements in PR compared to the findings in the placebo group. In the intestinal microbiota assessment, herbal medicine and probiotics synergistically increased beneficial bacteria counts. *Conclusion*. Combination therapy with herbal medicine and probiotics appears to relieve overall IBS symptoms by synergistically increasing beneficial intestinal microbe counts.

## 1. Introduction

Irritable bowel syndrome (IBS) is a functional gastrointestinal disorder characterized by abdominal pain, discomfort, and bowel disturbances without any structural abnormality [1]. IBS may cause significant inconvenience to patients, impair their social functioning, and deteriorate their quality of life [2]. The involvement of various factors in the pathophysiology of IBS makes treatment more difficult [3]. Factors such as imbalances of intestinal microbiota and increased intestinal permeability have been identified as important elements in the pathophysiology of IBS [4, 5]. Therefore, therapeutic approaches aimed at resolving disturbances in the intestinal microbiota and maintaining mucosal barrier homeostasis can be helpful in the treatment of IBS. However, due to dissatisfying results with conventional IBS treatments, complementary therapies including herbal medicine and probiotics are becoming attractive options for many patients [6].

Herbal medicines have long been used in Asian countries due to their safety and having only a few side effects. *Gwakhyangjeonggisan* (GJS; *Kkako-shoki-san* in Kampo Medicine; *Huoxiang-zhengqi-san* in Traditional Chinese Medicine), a study agent in this trial, has been found to relieve abdominal pain, diarrhea, and vomiting as an over-the-counter or prescribed medicine [7–9]. However, the clinical evidence supporting the efficacy of herbal medicine against IBS is weak [10]. Duolac7S (DUO), a probiotics mixture, has been also reported to have beneficial effects on IBS in a previous study [11]. Recently, the simultaneous administration of herbal medicines and probiotics has become a popular treatment for IBS in Korea [12]. However, no clinical studies have investigated the effect of combination therapy with herbal medicine and probiotics on IBS. In the present study, we evaluated and compared the effect of GJS combined with a multistrain probiotic mixture (DUO) on diarrhea-dominant IBS (D-IBS) symptoms to that of a placebo. In addition, intestinal permeability was assessed, and common strains of bacteria in the intestine were quantified using denaturing gradient gel electrophoresis (DGGE) and reverse transcription-polymerase chain reaction (RT-PCR) for mechanism analysis.

## 2. Materials and Methods

### 2.1. Participants

Sixty-four participants who met the criteria of D-IBS based on Rome III [1] were recruited at Kyung Hee University Hospital at Gangdong in Seoul. The inclusion and exclusion criteria for this study are shown in the protocol paper [13].

### 2.2. Study Protocol

**Figure 1:**
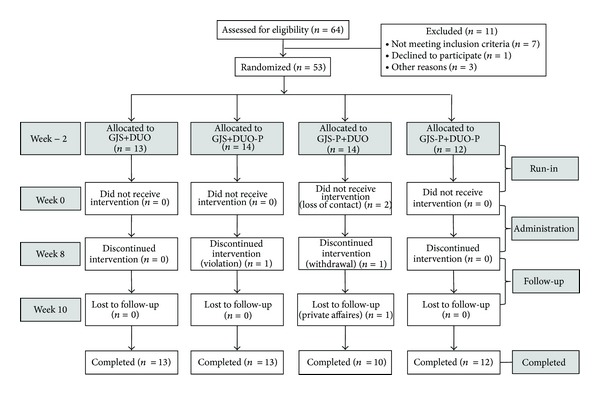
Flow chart of trial. GJS: *Gwakhyangjeonggisan*, DUO: Duolac7S, GJS-P: placebo of *Gwakhyangjeonggisan*, DUO-P: placebo of Duolac7S.

The current study was conducted as a double-blind, placebo-controlled trial with 64 participants being randomly allocated to 1 of the following 4 groups: (1) the real GJS and real DUO group (GJS+DUO), (2) the real GJS and placebo DUO group (GJS+DUO-P), (3) the placebo GJS and real DUO group (GJS-P+DUO), and (4) the placebo GJS and placebo DUO group (GJS-P+DUO-P). The participants completed a 2-week run-in (weeks −2 to 0), 8 weeks of administration (weeks 0–8), and a 2-week follow-up period (weeks 8–10). During the administration period, participants were to take 1 pack of GJS or its placebo 3 times a day (2 h after each meal) and 1 capsule of Duolac7S or its placebo 2 times a day (2 h after breakfast and dinner). Randomization was performed after an independent statistician screened participants by using random allocation numbers from a random number creation program. The investigator, clinical research coordinator (CRC), clinical pharmacist, and participants were blinded to randomization until the end of the study. We calculated the sample size based on previous similar studies [14, 15], as the study agent had never been studied, and determined that 64 participants were necessary because a sample size of 48 was regarded as the minimal number of participants needed for clinical significance, assuming a 25% dropout rate. This sample size provided 80% power to demonstrate the superiority of the study agents to placebos. The flow of the entire trial is described in Figure 1.

Informed consent was properly acquired prior to the trial at week −2. Participants were required to record their daily symptoms, responses to medication, and potential adverse effects. Safety was examined by blood testing at the end of the administration period (week 8). Compliance was calculated by determining the amount of medication returned, with usage of more than 80% of the medicine considered the minimum level of compliance. The protocol of the trial was approved by the institutional review boards and ethics committee at Kyung Hee University Hospital at Gangdong. The CRC assessed the study variables, and the entire procedure of the trial was monitored by an authorized clinical research organization, Marinet Corporation, Seoul, Korea. The detailed protocol of this trial was previously described [13].

### 2.3. Interventions

The GJS used in the trial was a brown, bitter, herbal extract granule (Gwakjungsan granule, Hanpoong Pharm & Food Co., Ltd., Jeonju, Korea) produced according to Korean Good Manufacturing Practice. Gwakjungsan granule (GJG), a water-extracted GJS combined with starch and lactose, was approved by the Korean Food & Drug Administration. GJG is composed of the 13 herbs [13]. Placebo GJG, which consisted primarily of cornstarch powder, has a similar color and taste as real GJG. Real GJG and its placebo were identically packed and sealed in the same opaque aluminum bags with the same labeling.

DUO (Cell Biotech Co., Ltd., Gimpo, Korea) is a probiotic mixture containing multiple species of 3 viable bacterial genera: 3 strains of *Bifidobacterium* (*B. brevis*, *B. lactis*, and* B. longum*), 3 strains of *Lactobacillus* (*L. acidophilus, L. plantarum, *and* L. rhamnosus*), and 1 strain of *Streptococcus* (*S. thermophilus*). Each capsule of DUO contains 5 billion bacteria (approximately 700 million bacteria for each strain). Placebo DUO, a powder consisting of cornstarch with a similar color and taste as DUO, was packaged in the same capsule to prevent it from being distinguished from real DUO.

### 2.4. Outcome Assessments

#### 2.4.1. Primary Outcome

Adequate relief (AR) was used as a primary outcome to assess the improvement of abdominal pain and discomfort. Patients were asked the following question on a weekly basis [14, 16]: “In the past 7 days, have you had adequate relief of your IBS pain or discomfort?” AR was measured from the end of each week during the run-in, administration, and follow-up periods. The proportion of responders (PR) was defined as the proportion of patients with at least 50% reductions of IBS pain and discomfort from week 0 to week 8.

#### 2.4.2. Secondary Outcomes

Patients were required to complete a diary to investigate the severity of the individual symptoms (abdominal pain, abdominal discomfort, bloating, flatulence, urgency, and mucus in the stool) and the severity of the overall symptoms on a 100 mm visual analog scale (VAS) during the entire trial [16]. On a daily basis, stool frequency, bowel functions according to the Bristol scale [17], and the ease of passage ranging from manual disimpaction to incontinence [18] were investigated. A quality of life questionnaire for persons with IBS (IBS-QoL), which was developed and validated by Drossman et al. [19] and translated into the Korean language [20], was used in this trial. The IBS-QoL consists of 8 dimensions including dysphoria, interference with activity, body image, health worry, food avoidance, social reaction, sex, and relationships. Each item is scored on a 5-point Likert scale, with a higher score representing a better quality of life [19]. The IBS-QoL was completed at weeks 0, 8, and 10.

#### 2.4.3. Intestinal Permeability

Intestinal permeability can be calculated by evaluating the urinary excretion of orally administered lactulose and mannitol [21]. The lactulose/mannitol (L/M) ratio can reflect the degree of intactness of the intestinal mucosal barrier, which plays a key role in the maintenance of normal intestinal function [22]. An increased L/M ratio is considered more likely to indicate digestive problems such as diarrhea due to leaky gut syndrome [22] or IBS [5, 23].

After an overnight fast, participants were asked to empty their bladders and then ingest lactulose and mannitol dissolved in water. Urine was collected over the next 8 h, and participants drank approximately 2 L of water during the test. Other food or liquid was not allowed. The collected 45 mL urine samples were stored in a −70°C deep freezer until analysis. Urinalysis was performed at weeks 0 and 8.

#### 2.4.4. The Species and Quantities of Intestinal Microbiota

The IBS symptoms of patients are closely associated with the presence or quantities of certain gastrointestinal bacteria [24]. The quantities of 7 bacterial species (*B. longum*,* B. brevis, B. lactis, S. thermophilus, L. rhamnosus, L. plantarum*, and* L. acidophilus*) and the *Firmicutes*/*Bacteroidetes* ratio were assessed. Participants collected their fecal samples for microbial analysis at weeks 0 and 8. The samples were sent to the laboratory in refrigerated containers and preserved at −70°C until analysis. The fecal samples were analyzed by an equipped laboratory (Cell Biotech Co., Ltd., Seoul, Korea) using DGGE and RT-PCR [25]. The detailed methods of DGGE and RT-PCR used in this trial were described briefly elsewhere [11].

### 2.5. Statistical Analysis

All data were collected and handled by an independent statistician. Researchers, clinicians, and investigators were rigorously isolated from data and data analysis until the last participant completed the trial. Both the *intention-to-treat* and *per-protocol* populations were analyzed. Pearson's chi-square test and analysis of variance (ANOVA) were used for baseline characteristics analysis. We selected the following factors as target variables for analysis in this trial: (1) AR and PR (primary outcomes), (2) VAS for abdominal pain and diarrhea, (3) bowel function scores (frequency, consistency, and ease of passage), (4) severity of individual symptoms (abdominal pain, abdominal discomfort, bloating, flatulence, urgency, and mucus in stool) and overall symptoms, and (5) IBS-QoL. Data are presented as the mean ± SD. Categorical variables were compared using the chi-square test or Fisher's exact test, whereas continuous variables were analyzed by ANOVA. All statistical analyses of the data were performed using the SPSS program, version 16.0 (SPSS Inc., Chicago, IL), and a *P* value <0.05 was regarded as statistically significant.

## 3. Results

### 3.1. Demographic Characteristics and Baseline Symptoms

Between April 2011 and February 2012, 64 patients were screened; of these, 11 participants failed to meet the inclusion criteria (Figure 1). Fifty-three patients (83% of the total enrollment number) were enrolled and randomly assigned to 1 of 4 groups (GJS+DUO, GJS-P+DUO, GJS+DUO-P, and GJS-P+DUO-P). Forty-eight (91%) participants completed the study. Five patients (9% dropout ratio) were dropped from the study due to protocol violations, consent withdrawal, private affairs, and loss of contact (Figure 1). Baseline demographic characteristics, the proportion of AR questions answered, bowel function scores, the severity of individual symptoms, overall symptoms, and quality of life were balanced among the 4 groups at the beginning of the study (Table 1).

### 3.2. AR and PR

**Figure 2:**
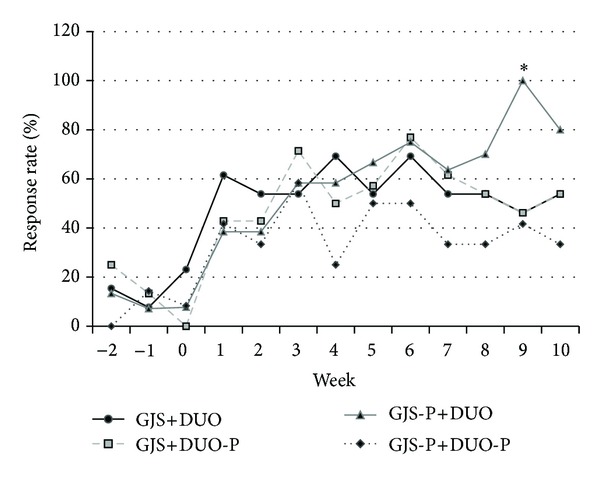
The weekly adequate relief (AR) among all eligible patients using the intention-to-treat principle, according to treatment group assignment. The response rate is the proportion of AR among patients in each group. The proportion of AR showed no statistically significant difference among 4 groups. AR: adequate relief, GJS: *Gwakhyangjeonggisan, *DUO: Duolac7S, GJS-P: placebo of *Gwakhyangjeonggisan*, DUO-P: placebo of Duolac7S, *P < 0.05.

**Figure 3:**
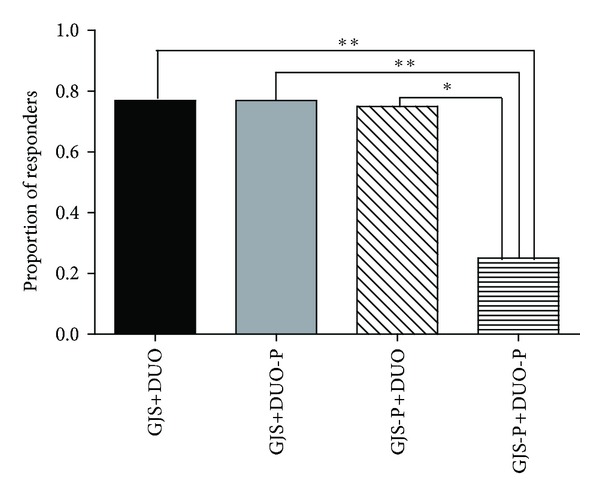
Proportion of responders^†^ among all eligible patients using the intention-to-treat principle, according to treatment group assignment. Compared with GJS-P plus DUO-P group, GSJ plus DUO group, GJS-P plus DUO group, and GJS plus DUO-P group showed statistically significant improvement during 8 weeks of administration period. ^†^Proportion of responders: proportion of patients who showed adequate relief of overall irritable bowel syndrome symptoms on at least half of the total weekly assessments (range: 0-1). GJS: *Gwakhyangjeonggisan*, DUO: Duolac7S, GJS-P: placebo of *Gwakhyangjeonggisan*, DUO-P: placebo of Duolac7S, *P < 0.05, **P < 0.01.

The proportions of AR questions answered tended to increase in all groups during the treatment and follow-up periods, but no significant differences were observed among the 4 groups (Figure 2). PR showed statistically significant improvement in the GJS+DUO, GJS-P+DUO, and GJS+DUO-P groups compared with the findings in the GJS-P+DUO-P group (Figure 3).

### 3.3. Bowel Function Scores, Individual Bowel Symptoms, and Overall Symptom Severity

Bowel function scores, individual symptoms (abdominal pain, discomfort, bloating, flatulence, urgency, and mucus in stool), and overall symptoms were improved in all groups after the administration of the study agents. In particular, the ease of passage tended to improve substantially in the GJS+DUO and GJS-P+DUO groups relatively to the findings for the other groups, but the improvement was not significant. Other symptoms did not display any significant differences among the 4 groups at week 8 (Table 2).

### 3.4. Quality of Life

Quality of life tended to be improved in all groups after treatment compared to the findings at week 0. However, there were no significant differences among the 4 groups before and after treatment. Moreover, when analyzed by the subcategories of quality of life (dysphoria, interference with activity, body image, health worry, food avoidance, social reaction, sex, and relationship), no significant differences among the 4 groups showed (Table 2).

### 3.5. Changes of the Quantities of Intestinal Microbiota in Feces and Intestinal Permeability Index (IPI)

**Figure 4:**
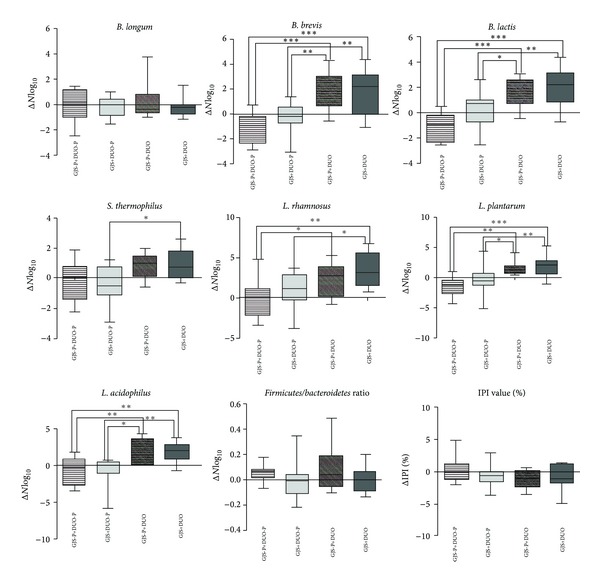
The change of 7 types of strains numbers in feces among 4 groups between week 0 and 8. Six types of strains (*B. brevis, B. lactis, S. thermophilus, L. rhamnosus, L. plantarum, and L. acidophilus*) showed significant difference after treatment. *Firmicutes*/*Bacteroidetes* ratio and IPI value did not show any statistically significant difference among 4 groups; however *Gwakhyangjeonggisan* plus placebo Duolac7S group tended to reveal lower value compared to other groups. Numbers of 7 types of bacterial strains are presented as log⁡_10_ average. Analysis was conducted by one-way ANOVA with Tukey's post hoc test. GJS: *Gwakhyangjeonggisan, *DUO: Duolac7S, GJS-P: placebo of *Gwakhyangjeonggisan*, DUO-P: placebo of Duolac7S, IPI: intestinal permeability index, *B. longum*: *Bifidobacterium longum*, *B. breve*:* Bifidobacterium breve*, *B. lactis*:* Bifidobacterium lactis*, *S. thermophilus*:* Streptococcus thermophilus*, *L. rhamnosus*:* Lactobacillus rhamnosus*, *L. plantarum*:* Lactobacillus plantarum*, *L. acidophilus*:* Lactobacillus acidophilus*, *P < 0.05, **P < 0.01, ***P < 0.001.

After the completion of treatment, there were significant changes in the quantities of intestinal microbiota in the patients' feces. Excluding *B. longum*, the quantities of all bacterial species were significantly different among the 4 groups (Table 3). As shown in Figure 4, combination therapy with GJS with DUO synergistically increased the quantities of 6 bacterial species: *B. brevis*, *B. lactis*, *S. thermophilus*, *L. rhamnosus*, *L. plantarum*, and* L. acidophilus*. In particular, significantly better effects were confirmed with GJS+DUO than with GJS+DUO-P or GJS-P+DUO when *B. lactis, L. rhamnosus*, and* L. plantarum* were investigated. Although the *Firmicutes*/*Bacteroidetes* ratio and IPI were not significantly different among the 4 groups, the GJS+DUO-P group displayed the greatest changes in the *Firmicutes*/*Bacteroidetes* ratio among the 4 groups (Figure 4).

### 3.6. Adverse Events

There were minor adverse events noted upon treatment with the study agents, including headache (2 patients), low-back pain (1 patient), constipation (1 patient), and dysmenorrhea (1 patient). However, these symptoms were all mild, and no serious adverse events were detected after 1 or 2 weeks of observation.

## 4. Discussions

A recent meta-analysis confirmed the effect of probiotics on alleviating some of the symptoms of IBS and improving quality of life [26]. A comprehensive review of medicine also demonstrated a benefit in managing IBS [27]. One of the promising probiotic mixtures for relieving IBS symptoms [11], DUO, and a representative herbal formula used for managing diarrhea and abdominal pain [7, 8], GJS, are currently administered simultaneously in many traditional Korean clinics. By investigating changes of symptoms and analyzing the mechanisms of IBS, we assumed that the combination intake of GJS and DUO might exert synergetic effects on the symptoms of IBS. As mechanism assessments, changes in the quantities of intestinal microbiota, and intestinal permeability were selected.

AR, as a primary outcome, displayed a tendency to increase in all groups after treatment compared to the findings before treatment; however, GJS or DUO did not significantly improve AR rates compared to the effects of placebo. The statistically significant AR at week 9 in the GJS-P+DUO group might be due to small sample size and might lack clinical meaning. Similar to our study, 2 previous studies utilized AR as a primary outcome for IBS. Neither Kim et al. [16] nor Leung et al. [28] reported any significant change of AR with the use of a probiotic mixture (VSL #3) and Chinese herbal medicine, respectively. The results of these studies may be explained by several reasons. First, AR does not reflect the complexity of the changes of IBS symptom due to its simplicity, and because patients depend on their memories to answer AR question, recall bias is possible. Additional studies using modified scales compensating for these defects are needed. The other potential cause was the placebo effect observed in this study. Approximately 40% of patients in the GJS-P+DUO-P (placebo group) reported symptomatic improvement after the treatment period (week 8). The placebo effect in this study corresponds to that of recent industry-generated drug studies with large numbers of patients, whose placebo response rate tended to be stable at 45% [29], whereas wider range of fluctuation (7–75%) of placebo response rates was observed in IBS studies with smaller sample size [30, 31]. Moreover, in a recent study, the open-placebo group showed a significantly greater improvement of AR than the waiting group [32]. This higher placebo response rate usually arises from regression to the mean, natural history, and Pavlovian conditioning in IBS [33]. In this study, the higher placebo response rate might be associated with the relatively long duration of the study (13 weeks), the high numbers of visits (13 visits including telephone visit), Pavlovian conditioning, augmented relationships with doctors, and a small sample size. Thus, additional studies using a waiting list group with larger sample sizes and excluding augmented relationships between doctor and patient are necessary in the future.

Despite the aforementioned limitations of AR, the PR assessment revealed the superiority of GJS, DUO, and the combination of GJS+DUO to placebo. Unlike AR, which is based on measurement periods of 1 week, PR evaluates the entire treatment period. Consequently, over the entire treatment period, GJS and DUO improved the major symptoms of IBS.

Our results revealed a lack of association between improvements of PR and individual IBS symptoms/IBS-QoL. The effect of probiotics and herbal medicine on IBS symptoms is controversial. Some studies demonstrated that probiotics have favorable effects on IBS symptoms [29, 34, 35], whereas others did not prove the superiority of probiotics to placebo [36, 37]. In addition, some studies of Chinese herbal medicine also reported improvement of IBS-related symptoms or stool form in the study group [38, 39], whereas others found no significant differences between the study and placebo groups [28, 40]. Recent studies assessing the effects of DUO or herbal medicine on quality of life reported no statistical differences between the study and placebo groups [11, 28]. In this study, although GJS and DUO did not confer a benefit in terms of individual symptoms and quality of life, the influence of GJS and DUO on IBS is still controversial. Thus, further research with a more elaborate design and large populations is needed.

Two previous studies reported lower quantities of *Lactobacillus* and *Bifidobacterium* populations in patients with IBS [41, 42]. One recent study evaluating intestinal microbiota reported that the administration of probiotics containing live *Bifidobacterium*, *Lactobacillus*, and *Enterococcus* populations could significantly increase *Bifidobacterium* and *Lactobacillus* counts in feces with IBS symptom improvement [43]. Therefore, it can be postulated that the increase in quantities of *Lactobacillus* and *Bifidobacterium* species by probiotics induces beneficial effects for patients with IBS. Based on the result of the composition of intestinal microbiota, our study indicated that combination therapy with DUO and GJS has synergetic effects on the human intestinal microbiota. This suggested that GJS serves as a prebiotics and efficiently supports the proliferation of beneficial bacteria in the human intestine. As the *Firmicutes*/*Bacteroidetes* ratio of patients with IBS is significantly higher than that of healthy controls [44], the fact that the GJS+DUO-P group tended to have a lower *Firmicutes*/*Bacteroidetes* ratio than the other groups might illustrate the effect of GJS alone on regulating harmful bacteria in the human intestine.

The study had certain limitations: the beneficial effect of GJS or DUO on intestinal microbiota did not reflect the individual symptoms. In addition, due to the multifactorial nature of IBS, the development of IBS cannot be explained by intestinal microbe changes alone. Concurrent treatment for IBS targeting several causes more effectively managed IBS symptoms. However, the possibility that GJS+DUO is synbiotics might be identified in this study; therefore, GJS+DUO can be used as a complementary therapy for IBS. Further studies that investigate the influence of each ingredient of GJS on the intestinal human microbiota and control other mechanisms such as intestinal hypersensitivity or motility disturbance should be considered in the future.

According to previous studies, patients with IBS have a higher intestinal permeability than healthy controls, which could be a possible aggravating factor of IBS symptoms [21, 22, 45]. Although IPI change was not significantly different among the 4 groups, a decreasing tendency was observed in the GJS+DUO, GJS+DUO-P, and GJS-P+DUO groups compared with GJS-P+DUO + P group after administration period. A previous study noted that an L/M ratio >0.07 is considered abnormal [21], and additional analysis with only patients with abnormally high IPIs revealed more prominent decreases in the GJS+DUO and GJS+DUO-P groups without statistical significance. Considering this decreasing tendency and the comparatively small number of patients with abnormal IPIs, further studies targeting only participants with high IPIs are expected to clarify the effect of GJS, DUO, or combination therapy with GJS and DUO on this variable. Moreover, as one recent study reported GJS's function of protecting the intestinal barrier [46], the individual effect of GJS on the IPI needs to be investigated in the future.

## 5. Conclusions

GJS and DUO individually had effects on major IBS symptoms. Simultaneous administration of GJS and DUO did not show any significant effect on individual symptom severity and quality of life. However, simultaneous administration with GJS and DUO improved the quantities of beneficial bacteria more than the individual administration. Further large-scale studies with more than 12 weeks of long term design and various mechanism investigations that consider the multifactorial nature of IBS are needed.

## Figures and Tables

**Table 1:** Characteristics of the patients and the baseline adequate relief*, bowel function scores**, symptom scores**, and IBS quality of life scores**.

Variables	GJS+DUO (*n* = 13)	GJS+DUO-P (*n* = 14)	GJS-P+DUO (*n* = 14)	GJS-P+DUO-P (*n* = 12)	*P* value
Mean age (SD)	49.6 (14.3)	47.5 (13.6)	47.1 (10.5)	47.5 (16.0)	0.959
Mean BMI (SD)	22.8 (3.6)	22.3 (3.8)	22.9 (3.0)	23.3 (3.2)	0.684
Male (%)	73.3	62.5	50.0	76.5	0.415
Smoking (%)	20.0	25.0	18.8	17.6	0.974
Drinking (%)	80.0	56.3	62.5	52.9	0.423
Adequate relief (%)	15.4	25.0	13.3	0.0	0.243
Bowel function score					
Frequency	15.54 (8.60)	13.64 (7.07)	15.00 (7.08)	12.23 (5.42)	0.646
Consistency^†^	1.66 (1.02)	0.87 (0.61)	1.13 (0.76)	1.31 (0.91)	0.079
Ease of passage^‡^	1.04 (1.09)	1.19 (0.74)	1.30 (0.80)	1.21 (0.69)	0.326
Symptom score (mm)					
Abdominal pain	38.49 (20.40)	31.04 (17.73)	40.48 (17.60)	32.37 (22.24)	0.554
Abdominal discomfort	39.65 (20.17)	34.82 (19.72)	43.87 (17.54)	36.82 (17.44)	0.638
Bloating	39.11 (21.20)	30.12 (17.25)	40.35 (19.95)	35.47 (20.95)	0.554
Flatulence	36.04 (16.72)	27.41 (15.63)	38.57 (17.07)	32.96 (20.51)	0.400
Urgency	38.97 (18.34)	34.58 (18.73)	44.33 (14.59)	38.44 (13.62)	0.507
Mucus in stool	36.65 (20.83)	26.43 (18.28)	37.50 (17.77)	35.31 (19.64)	0.416
Overall symptom	46.90 (23.24)	36.77 (18.24)	51.08 (12.98)	43.40 (14.15)	0.209
Overall IBS quality of life	52.00 (29.14)	42.21 (22.49)	54.92 (24.78)	41.00 (18.26)	0.366

GJS: *Gwakhyangjeonggisan*, DUO: Duolac7S, GJS-P: placebo of *Gwakhyangjeonggisan*, DUO-P: placebo of Duolac7S.

Baseline values were analyzed by Fisher's exact test for categorical variables and one-way ANOVA for continuous variables.

*Adequate relief is presented as the percentage of the number answering “yes” to AR question/total number.

**Bowel function, symptom, and IBS quality of life scores are presented as mean (standard deviation).

^†^Used a scale of 1–7: 1, watery stool; 7, hard, lumpy stool (based on Bristol stool form scale).

^‡^Used a scale of 1–7: 1, fecal incontinence; 7, manual disimpaction.

**Table 2:** Bowel function scores, symptom scores, and IBS quality of life scores after administration of study agents (8 weeks, intention-to-treat analysis).

Variables	GJS+DUO (*n* = 13)	GJS+DUO-P (*n* = 14)	GJS-P+DUO (*n* = 14)	GJS-P+DUO-P (*n* = 12)	*P* value
Bowel function score					
Frequency	14.31 (7.38)	14.00 (8.93)	12.25 (7.30)	12.00 (4.97)	0.806
Consistency^†^	1.19 (0.93)	0.96 (0.65)	1.15 (1.06)	0.85 (0.69)	0.862
Ease of passage^‡^	1.21 (0.73)	0.80 (0.61)	1.32 (1.04)	0.82 (0.73)	0.108
Symptom score (mm)					
Abdominal pain	28.33 (20.60)	27.97 (18.35)	28.45 (18.55)	24.61 (14.87)	0.947
Abdominal discomfort	31.71 (19.44)	29.93 (17.51)	29.43 (19.78)	27.39 (14.26)	0.945
Bloating	26.08 (16.11)	25.65 (15.10)	31.81 (18.40)	28.73 (13.83)	0.752
Flatulence	25.67 (16.70)	23.88 (16.07)	28.27 (18.56)	31.60 (16.66)	0.689
Urgency	27.31 (21.53)	26.00 (19.83)	25.20 (15.23)	27.13 (15.58)	0.992
Mucus in stool	25.60 (23.75)	21.81 (19.66)	23.06 (16.70)	28.18 (19.21)	0.856
Overall symptom	32.37 (21.88)	30.98 (17.43)	31.55 (17.98)	33.65 (14.63)	0.985
IBS quality of life					
Dysphoria	8.92 (7.69)	9.29 (7.69)	10.08 (6.29)	10.42 (6.57)	0.948
Interference with activity	8.85 (7.45)	7.00 (4.61)	8.38 (5.01)	7.92 (6.56)	0.871
Body image	3.92 (3.59)	2.57 (3.39)	4.00 (2.90)	2.92 (2.57)	0.586
Health worry	3.62 (2.43)	3.29 (2.09)	3.85 (1.91)	4.00 (2.26)	0.848
Food avoidance	5.46 (2.50)	5.50 (3.20)	5.25 (2.49)	4.17 (3.43)	0.641
Social reaction	4.38 (4.75)	3.50 (3.35)	4.09 (3.08)	3.92 (3.34)	0.938
Sexual	1.46 (1.94)	1.43 (1.95)	1.58 (2.07)	1.67 (1.61)	0.988
Relationship	2.62 (2.79)	2.29 (1.73)	3.15 (2.30)	2.42 (2.31)	0.781
Overall	39.23 (30.43)	34.86 (24.14)	39.85 (22.97)	37.42 (24.43)	0.958

GJS: *Gwakhyangjeonggisan*, DUO: Duolac7S, GJS-P: placebo of *Gwakhyangjeonggisan*, DUO-P: placebo of Duolac7S.

All values were analyzed by Fisher's exact test for categorical variables and one-way ANOVA for continuous variables.

All scores are presented as mean (standard deviation).

^†^Used a scale of 1–7: 1, watery stool; 7, hard, lumpy stool (based on Bristol stool form scale).

^‡^Used a scale of 1–7: 1, fecal incontinence; 7, manual disimpaction.

**Table 3:** The change of 7 types of strains numbers, *Firmicutes/Bacteroidetes* ratio in feces, and IPI value among 4 groups between 0 week and 8 weeks.

	Variables	GJS-P+DUO-P (*n* = 13)	GJS+DUO-P (*n* = 13)	GJS-P+DUO (*n* = 10)	GJS+DUO (*n* = 12)	*P* value
Δ*N* log_10_ (Mean ± SD)/1 g feces of bacterial species	*B. longum *	−0.016 ± 0.424	−0.147 ± 0.203	0.302 ± 0.448	−0.184 ± 0.198	0.695
	*B. brevis *	−1.120 ± 0.405	−0.333 ± 0.282	1.850 ± 0.477	1.670 ± 0.485	**<0.001**
	*B. lactis *	−0.993 ± 0.357	0.177 ± 0.369	1.811 ± 0.381	2.060 ± 0.412	**<0.001**
	*S. thermophilus *	−0.168 ± 0.402	−0.388 ± 0.320	0.802 ± 0.252	0.842 ± 0.270	**0.01**
	*L. rhamnosus *	−0.553 ± 0.788	0.654 ± 0.678	2.478 ± 0.682	3.525 ± 0.592	**<0.001**
	*L. plantarum *	−1.338 ± 0.465	−0.324 ± 0.538	1.528 ± 0.351	2.029 ± 0.463	**<0.001**
	*L. acidophilus *	−0.784 ± 0.601	−0.537 ± 0.447	1.723 ± 0.550	1.790 ± 0.370	**<0.001**
	*Firmicute/Bacteriodetes *	0.410 ± 0.149	−0.139 ± 0.226	0.392 ± 0.313	−0.050 ± 0.199	0.211

The change of IPI value		0.136 ± 2.025	−0.546 ± 1.642	−1.190 ± 1.413	−0.741 ± 1.980	0.398

GJS: *Gwakhyangjeonggisan*, DUO: Duolac7S, GJS-P: placebo of *Gwakhyangjeonggisan*, DUO-P: placebo of Duolac7S, IPI (%): intestinal permeability index (Lactulose/mannitol).

The change of number of bacterial species was analyzed by one way-ANOVA and presented as Δ*N* log_10_ (mean ± SD)/1 g feces.

The change of intestinal permeability index is presented as percentage (%).
